# Axon Guidance Molecules in the Islets of Langerhans

**DOI:** 10.3389/fendo.2022.869780

**Published:** 2022-04-14

**Authors:** Bayley J. Waters, Barak Blum

**Affiliations:** Department of Cell and Regenerative Biology, University of Wisconsin, Madison, WI, United States

**Keywords:** islets of Langerhans, islet architecture, islet morphogenesis, axon guidance molecules, slit-robo, semaphorin-neuropilin, ephrin-eph, netrins

## Abstract

The islets of Langerhans, responsible for regulating blood glucose in vertebrates, are clusters of endocrine cells distributed throughout the exocrine pancreas. The spatial architecture of the different cell types within the islets controls cell-cell communication and impacts their ability to collectively regulate glucose. Islets rely on a range of chemotactic and adhesive cues to establish and manage intercellular relationships. Growing evidence indicates that axon guidance molecules such as Slit-Robo, Semaphorin-Neuropilin, Ephrin-Eph, and Netrins, influence endocrine progenitors’ cell migration to establish correct architecture during islet morphogenesis, as well as directly regulating physical cell-cell communication in the mature islet to coordinate hormone secretion. In this mini-review, we discuss what is known and not yet known about how axon guidance molecules contribute to islet morphogenesis and function.

## Introduction

The islets of Langerhans, endocrine mini-organs distributed throughout the pancreas, regulate blood glucose in vertebrates by tightly coordinated hormone secretion. The spatial architecture of endocrine cell types within the islet is important to their function, as physical contact and secreted signals between certain cell types optimizes hormone secretion (1). While islet architecture varies across vertebrate species, homotypic interactions among cell types are generally conserved (2), supporting the idea that same cell contacts serve a functional purpose in the islet. For example, β cells which touch one another are able to synchronize insulin secretion, promoting coordinated insulin release (3). Islet architecture is also observed to be disrupted in diabetic mouse models and human diabetic patients (4), highlighting the relationship between islet morphology and islet function.

During development, islet cells differentiate into their mature identities, concurrently migrating to their final locations within the pancreas and arranging themselves into their mature cytoarchitecture (5). Islets utilize a variety of adhesive and chemotactic cues to regulate these morphogenic activities. Many of these signaling cues are also known to assist in cell-cell communication and hormone secretion in mature islets. This indicates that the molecules serve dual and potentially related roles in establishing islet morphology and assisting in islet function throughout life.

Axon guidance molecules are one key class of chemotactic signaling cues. Originally named for their essential role in neuronal migration, branching, and synapse formation within the central nervous system (CNS) (6), research continues to reveal diverse activity of axon guidance molecules in many other tissues of the body. Among other roles, axon guidance molecules have been shown to be involved in lung branching morphogenesis, ureteric bud formation in the kidney, endothelial cell migration during cardiovascular development, and mammary gland outgrowth, as discussed in a prior review (7).

A growing body of research has demonstrated that axon guidance molecules Slit-Robo, ephrin-Eph, semaphorin-neuropilin, and netrins are also essential in both the morphological development and secretory function of islets of Langerhans. Below, we review the known and potential activities of these islet-expressed axon guidance molecules in building the functional islet during morphogenesis, and in maintaining the cell-cell connectivity essential to proper islet function in adulthood.

## Axon Guidance Molecules in Islet Morphogenesis

Islet development and morphogenesis begin when endocrine progenitors of epithelial origin delaminate and migrate away from pancreatic ducts and across the pancreatic mesenchyme (5, 8–10). During morphogenesis, early islet cells must receive chemotactic cues to migrate and aggregate into clusters. Defects in islet cell migration can result in islets that do not appropriately migrate away from the ducts, or do not form cell clusters with proper architecture. Certain axon guidance molecules, namely semaphorin-neuropilin, ephrin-Eph, Slit-Roundabout, and netrins, have been shown to play vital roles in these aspects of islet morphogenesis.

### Semaphorin and Neuropilin

Endocrine progenitors begin to delaminate and migrate away from the ducts around embryonic day (E)14.5 in the mouse (11, 12), driven in part by chemotactic cues by the axon guidance proteins, semaphorins, and their receptors, neuropilins. Neuropilin 2 (Nrp2) is expressed in islet endocrine cells as early as E15.5. At E15.5, *Nrp2* whole body knockout mice exhibited improper migration of endocrine cells away from the ductal structures and instead had long strings of cells lining the ducts. At postnatal day (P)1, when wildtype mice showed evidence of nascent islets with expected β cell core and α cell mantle distributed throughout the pancreas, *Nrp2* mutant mice showed large aggregates of endocrine cells surrounding the ductal structures. Quantification revealed that *Nrp2* mutants had significantly reduced distance between ducts and islets at both developmental timepoints, a relationship which was also observed in adulthood (13). These data indicate that Nrp2 is required for proper islet cell migration away from the ducts during morphogenesis, with lasting impact on the location of islets in adulthood.

**Figure 1:**
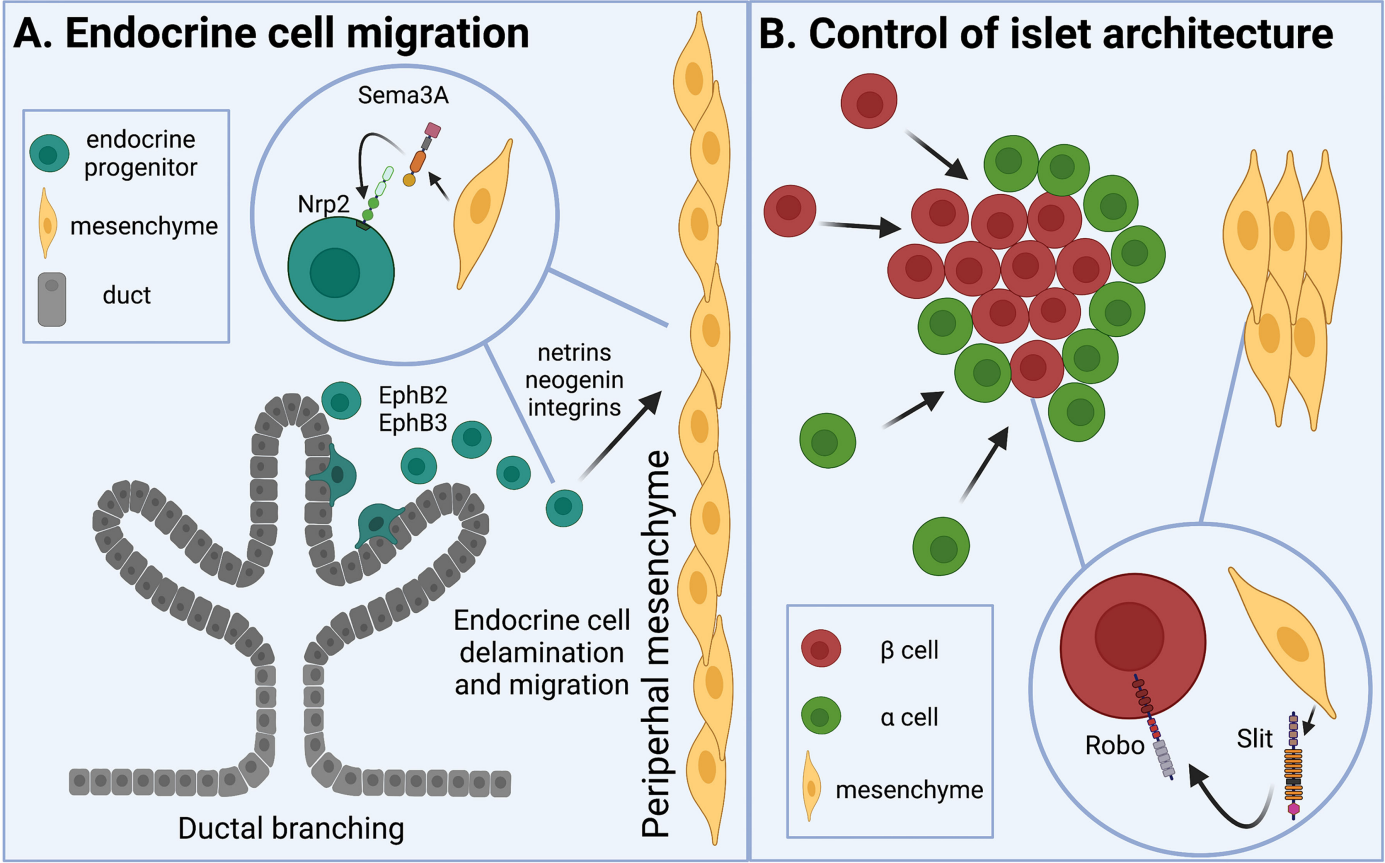
Axon guidance molecules are involved in islet morphogenesis and cell-cell contact. **(A)** Islet development begins as proendocrine cells delaminate from branching ducts and migrate across the pancreatic mesenchyme. Eph receptors are involved in epithelial cell branching. Semaphorins expressed by peripheral mesenchyme draw neuropilin receptor-expressing proendocrine cells away from the ducts. Netrins, neogenin, and integrins are also known to regulate epithelial cell migration. **(B)** As endocrine cells aggregate into islets, Slit from pancreatic mesenchyme interacts with Robo on β cells to direct cell type sorting and establish appropriate islet architecture.

Mesenchyme-derived Semaphorin3a (Sema3a) likely signals through Nrp2 to regulate this endocrine cell migration within the pancreas. Sema3a is expressed in the peripheral pancreatic mesenchyme during development (13). In fetal (E13.5) mouse pancreatic explants grown *in vitro*, β cells migrated toward Sema3a-soaked beads (13). Therefore, peripheral mesenchyme may secrete Sema3a which is able to signal through Nrp2 on endocrine progenitors to guide them through mesenchymal tissue and away from the ducts (**Figure 1A**).

Neuropilins are reported to act together with their coreceptors, plexins; however, direct evidence that plexins are involved in islet cell migration has not yet been uncovered. Various plexin family members are expressed in the developing mouse pancreas. PlexinB1 is expressed in epithelial duct cells between E13.5-E15.5 (14), during the time of endocrine cell delamination. PlexinA3 is also enriched in fetal pancreatic endocrine cells at E15.5 (13). The presence of plexin family members in these cell types at these key embryonic timepoints suggests that plexins could be involved in the Sema-Nrp-controlled endocrine cell migration during pancreas development.

### Ephrin and Eph

Most of the known impact of ephrin-Eph signaling in the islet is understood in the context of hormone secretion in mature islets. However, there is evidence that this signaling pair may play a role in islet development. EphB2/EphB3 receptors are required for pancreatic epithelial branching. Subsequent to observed defects in branching, *EphB2/EphB3* knockout mice also had fewer endocrine cells in development and adulthood (15). EphB3 was also found to be transiently expressed during development and marked delaminating epithelial endocrine-committed cells (16).

In addition to being implicated in epithelial branching and proendocrine cell identity, a new study has revealed a potential role of ephrin-Eph signaling in establishing islet architecture *via* changes in Notch signaling. RNA sequencing of islets from a gain-of-function Notch mutant mouse revealed an increase in ephrin signaling. In dispersed islet cells, treatment with soluble ligand ephrinA5-Fc increased repulsion among β cells and blocked pseudo-islet formation (17). Together, these data show that ephrins and Ephs may be involved in both endocrine cell differentiation and endocrine cell migration. Further understanding of the mechanisms by which Ephs and ephrins mediate these processes would be an important focus of future research.

### Slit and Roundabout

Axon guidance molecules Slit and Robo are involved in early pancreatic differentiation. In a mesenchyme-specific Pbx1 deletion model, which showed significant reduction in pancreatic proendocrine cells during development, Slit expression was also reduced; exogenous Slit applied to pancreatic explants was sufficient to rescue the number of insulin positive cells (18). Similarly, Robo is required for pancreatic progenitors to maintain endocrine identity (19). Both studies highlight roles of mesenchymal Slit and epithelial Robo in islet development.

As early endocrine cells migrate within the pancreas, they also begin to cluster together to form the islets. The nascent endocrine cells start to differentiate and mature into their final identities as β, α, δ, ϵ, and PP cells. In many animals, including mice and humans, these proendocrine cells will arrange themselves spatially to prioritize β cell-β cell interactions, resulting in clusters of homotypically-interacting β cells surrounded by other endocrine cell types (2, 20). In mouse islets, this manifests as a core of β cells in the center of the islet, surrounded by other endocrine cell types on the islet periphery, or mantle. Recent work has also identified the importance of mesenchymal Slit and epithelial Robo in the formation of proper spatial architecture, or cell type arrangement, in the islet. Robo expression in the β cell is required for appropriate endocrine cell-type sorting as the islet develops in prenatal and early postnatal life (21). Deleting *Robo* in the β cells, but not in the α cells, of developing mice results in cell type intermixing and loss of classic murine core-mantle architecture.

Slit may interact with Robo to facilitate its activity, but this has not been directly confirmed. While Slit1 is expressed in the islet during development, it is not required for endocrine cell type sorting, as whole-body *Slit1* null mice exhibit no noticeable abnormalities in islet architecture at any timepoint. Slit2 and Slit3 are only expressed in the pancreatic mesenchyme, around or prior to E18.5, and deletion of both, but not either of them separately, results in loss of proper islet formation (22). These data suggest that Slits 2 and 3 from the pancreatic mesenchyme may interact with Robo in the β cell to regulate endocrine cell migration and establish islet architecture (**Figure 1B**).

Robo is known or purported to have binding partners other than Slit. Recent evidence identified Neurexin (Nrxn), a co-receptor with Robo, as an essential member of a Slit-Robo-Nrxn complex that controls excitatory synapse formation in the developing hypothalamus. Specifically, presynaptic Nrxn interacts with postsynaptic Robo (23). All three Nrxn family members show expression in the fetal mouse pancreatic mesenchyme at E15.5 (24), providing substrates for potential mesenchymal Nrxn-endocrine Robo crosstalk in islet formation. Nrxn1 continues to be expressed in the adult islet, while Slit2 and Slit3 appear to be expressed in pancreatic stellate cells in the adult (25). It would thus be interesting to see if Nrxn also participates in Slit-Robo-mediated functions in the islet.

### Netrin

Netrins are known to be involved in epithelial morphogenesis in several tissues (26, 27). However, understanding of the activity of this axon guidance family in islet morphogenesis is limited. Netrin1 is expressed in fetal rat endocrine and exocrine pancreas during the time of islet morphogenesis, as well as in pancreata undergoing tissue remodeling (28), suggesting that it may be involved in crosstalk among these tissues. It has also been shown to impact the migration and adhesion of fetal epithelial cells (28, 29).

While the canonical netrin receptor, DCC, is not expressed in the pancreas, another receptor, neogenin, is expressed in fetal pancreas and in pancreas undergoing tissue remodeling (28). Netrin receptors integrins α6β4 and α3β1, which have also been shown to regulate epithelial cell migration, are expressed in the pancreatic epithelium during development (29). The expression of netrins and their receptors during development and regeneration, as well as the epithelial-mesenchymal expression of these binding pairs, suggests that netrins and their receptors could play some role in islet morphogenesis. Prolific evidence of netrins’ roles in epithelial tissue development coupled with the expression of netrins in the developing pancreas suggests that further research on netrins in islet development would be timely.

## Axon Guidance Molecules in Islet Function

There is ample evidence that the ways that cells within the adult islet communicate with one another are critical to their ability to regulate glucose. Islets display characteristic cytoarchitecture and cell-cell interactions that optimize their regulatory functions (4, 30). β cells collectively signal to each other to increase or decrease insulin secretion (31). β cells that are directly touching each other are able to electrically couple, namely *via* connexin36 gap junctions, to coordinate pulsatile insulin release (32). Therefore, understanding how cells within the islet make or maintain contact with one another can shed light on how islets optimize hormone secretion. Certain axon guidance molecules have been shown to directly or indirectly impact islet hormone secretion.

### Ephrin and Eph

Eph receptors are activated by membrane-bound ephrin ligands. Ephs and ephrins utilize bidirectional signaling, wherein ephrin ligands can signal *via* Eph receptors to induce intracellular signals downstream of Eph (Eph forward signaling), and Eph receptors can signal back through ephrins into the ligand host cell, inducing signals downstream of ephrin (ephrin reverse signaling) (33). Various studies have demonstrated that Eph-ephrin signaling plays a part in regulating hormone secretion in islets. Both homotypic β-β and heterotypic β-α cell contacts are regulated by ephrins. β cells communicate directly with one another *via* ephrinA5 ligand and EphA5 receptor, as evidenced by localization of both ligand and receptor on the touching surfaces of β cells (34).

**Figure 2:**
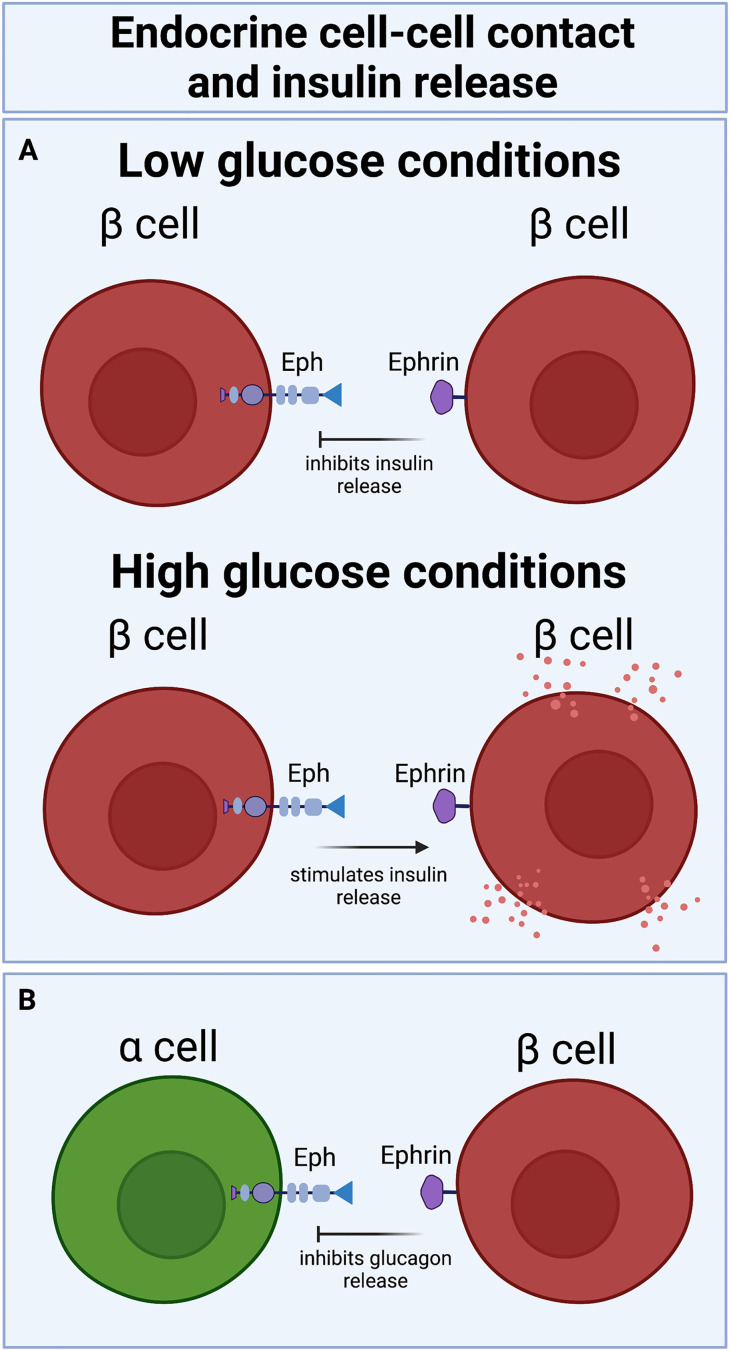
Bidirectional Eph-ephrin signaling increases or decreases insulin release in a glucose-dependent manner: **(A)** eprin-Eph forward signaling attenuates insulin secretion under basal conditions, while Eph-ephrin reverse signaling potentiates insulin secretion under high glucose conditions. **(B)** β cell-α cell ephrin-Eph signaling also decreases glucagon secretion.

How ephrin-Eph signaling impacts insulin secretion among β cells depend on the direction of the signaling. While EphA5 forward signaling decreases insulin secretion, ephrinA5 reverse signaling increases insulin secretion (**Figure 2A**). The balance between forward and reverse signaling is glucose dependent; under low glucose conditions, Eph forward signaling limits basal insulin secretion. Addition of glucose to the system dephosphorylates Eph receptor, decreases Eph forward signaling and promotes ephrin reverse signaling, resulting in increased glucose-stimulated insulin secretion (GSIS) (34).

In line with these findings, perturbing ephrin reverse signaling by deleting ephrinA5 in mouse islets reduced GSIS (34). Notably, stimulation of GSIS by ephrin reverse signaling among β cells also requires connexin36 gap junctions (34), indicating that ephrin-mediated GSIS necessitates that β cells be electrically coupled and synchronizing ion fluxes, as detailed in a prior review (35). Ephrins from β cells were also found to signal to EphA4 receptor on α cells to inhibit glucagon secretion (**Figure 2B**) by modulation of F-actin (36). Glucagon secretion is similarly regulated by β cell ephrinA5 and α cell EphA4/7 signaling in reaggregated pseudoislets (37).

In other studies, when ephrin-Eph forward signaling was blocked using an Eph receptor antagonist, GSIS increased in healthy mice (38) and in a diabetic mouse model (39). EphrinA5-Fc and EphA5-Fc fusion proteins have been shown to enhance β cell communication among dispersed islet cells and have thus been used to improve their survival in supplemented hydrogels, which are used to encapsulate β cells for increased protection in transplant (40). Ephrin-Eph signaling is also implicated in islet vasculature. The endothelial cells of islet blood vessels preferentially express EphA4 receptors in comparison to non-islet vasculature (41).

The mechanistic underpinnings of how Ephs and ephrins may regulate insulin secretion among β cells are beginning to be explored. EphrinA5-Fc and EphA5-Fc fusion proteins were shown to modulate the structural characteristics of F-actin, altering insulin secretory granule access to the plasma membrane (34). Ephrin-Eph signaling may also regulate insulin secretion by its activity in β cell cilia. Ciliary function in the β cell is required for proper insulin secretion and glucose tolerance (42). EphA2/3 are hyperphosphorylated in mutant mice with impaired β cell ciliary function, which also exhibit an increase in glucose intolerance and a decrease in insulin secretion over time. EphrinA5-Fc treatment reduced hyperphosphorylation of EphA2/3 and rescued insulin secretion in these mutant mice (43).

In human type 2 diabetic (T2D) donor islets, β cells showing markers of metabolic inflexibility also showed higher ephrin-Eph signaling activity, although details about the direction of signaling are not clear. Higher ephrinA5 expression in these T2D donor islets correlated with alterations in islet architecture (17), lending support to a connection between islet function and islet morphology. Interestingly, there seem to be some contradictions on the role of ephrins in β cell activity; in some studies, ephrin5A appears to improve β cell connectivity and function (40, 43), whereas in others, ephrin5A reduces β cell-β cell contacts and appears to negatively impact β cell function (17). This discrepancy may be due to the mechanisms behind ephrin-Eph signaling in the islet, such as phosphorylation status, or the directionality of ephrin-Eph signaling in each instance, which as illustrated above and in **Figure 2** can have opposing effects on hormone secretion. A better understanding of the functional consequences of ephrins and Ephs in the islet demands further investigation.

### Slit and Roundabout

Both Slit and Robo have been reported to play key roles in adult islet function. Exposing isolated islets to soluble Slits in their media increased insulin secretion (44). In addition, *Slit* knockdown in the murine β cell line MIN6, as well as in primary mouse islet cells, resulted in a dramatic decrease in cell survival; supplemented Slit3 or a mixture of all three Slit ligands rescued cell survival. Slits were also shown to protect β cells from stress-related death under high glucose conditions by reducing ER stress and apoptosis (44).

Deletion of *Robo* in mouse β cells during development impacts endocrine cell type sorting and disrupts proper islet architecture (21), resulting in an islet with reduced β cell-β cell contacts. Decreased homotypic β cell interactions attenuate synchronous insulin release; thus, loss of Robo indirectly contributes to less coordinated glucose regulation (45). Evidence suggests that the decrease in synchronous insulin release is not due to changes in endocrine cell number, vasculature, or innervation, nor is it caused by a decrease in connexin36 gap junction proteins on β cells (45), indicating that the decrease in coordinated insulin signaling seen in *Robo* mutant mice is a direct result of loss of homotypic β cell-β cell contact. Robo may thereby impact insulin secretion and islet function in adulthood by facilitating homotypic β cell contacts during development. Whether Robo continues to play a role in maintaining homotypic β cell interactions in adulthood has not yet been uncovered.

While the aforementioned evidence indicates Robo’s indirect role in β cell function by its modulation of homotypic β cell contacts in development, expression data show that Robo is also implicated in insulin-resistant or obesity-associated diabetes. In both mouse and human diabetes, *Robo* expression in the islet is decreased. Expression of Slit and Robo family members is decreased in isolated mouse islets subjected to variable stressors combined with high glucose (44). *Robo1* and *Robo2* are downregulated in the islets of *Lep^ob/ob^* mice, a common mouse model for obesity-associated diabetes (21). *ROBO1* and *ROBO2* are also downregulated in islets from obese and diabetic human donors, in comparison to non-diabetic controls (46–48).

### Netrin

Netrin expression has been identified in adult islets from mice and humans (49). The two most abundant netrins, Netrin-4 and Netrin-1, are expressed in a cell type-specific way, with Netrin-1 being expressed in mouse and human β cells, and Netrin-4 expressed in human α cells and in both α and β cells in mice. Netrin expression in the islet is associated with increased cell survival and decreased apoptosis. Exogenously supplied Netrin-1 and Netrin-4 decreased apoptosis in MIN6 cells under hyperglycemic conditions by degrading netrin receptors neogenin and Unc5, and thereby decreasing caspase3 cleavage (49). Netrins also induced Akt and Erk pro-survival signaling (49).

Netrin-1 treatment increased insulin secretion in mouse islets *ex vivo* and decreased fasting glucose levels in a diabetic mouse model *in vivo* (50). In line with this finding, Netrin-1 was shown to be significantly reduced in newly diagnosed type 2 diabetic (T2D) patients compared to healthy controls; Netrin-1 levels were inversely correlated with insulin resistance in these T2D patients (51). These data suggest that netrins are important for islet cell survival and insulin secretion. However, the mechanism(s) by which netrins may increase hormone secretory function of islet cells are not yet known.

## Concluding Remarks

Most data on axon guidance molecules focus on their activity in rodent islets or isolated human islets from healthy donors; however, there is evidence that some of these chemotactic molecules show changed expression in human diabetic patients. Ephrin-Eph signaling is altered in human T2D patients and corresponds with changes in islet architecture (17). Netrin-1 levels were reduced in the plasma of T2D patients, showing an inverse relationship with insulin resistance (51). Transcriptomic analyses reveal that *ROBO1* and *ROBO2* genes are differentially expressed in islets from T2D patients (46–48). Using cluster polygenic scores, *ROBO2* was also shown to be associated with human gestational diabetes (52).

The islets of Langerhans utilize highly organized architecture, which prioritizes physical cell-cell contacts that optimize intra-islet signaling, to carry out their glucoregulatory activities. Increasing focus on axon guidance molecules in islet morphogenesis and cell-cell communication have connected this class of chemotactic molecules to islet function. Semaphorin-Neuropilin and Slit-Robo binding partners have been shown to be essential in islet cell migration and cell type sorting during islet morphogenesis (**Figure 1**). Some research also implicates netrins and Eph-ephrin signaling in epithelial cell migration and branching in the pancreas. Robo and Eph-ephrin signaling are known to regulate β cell-β cell and β cell-α cell physical contact, thereby impacting cell-cell communication and hormone secretion from these cell type (**Figure 2**). Ephrins and netrins have also been shown to increase β cell survival and insulin secretion. Continued exploration of the roles of axon guidance molecules in islet function is a timely and important focus of islet biology and of diabetes research.

## Author Contributions

BB and BJW conceived, wrote, and edited this mini-review. All authors contributed to the article and approved the submitted version.

## Funding

This work was funded in part by grant number R01DK121706 from NIDDK to BB. BJW was funded by a UW-Madison Endocrinology-Reproductive Physiology Training Grant number T32 HD041921 from NICHD.

## Conflict of Interest

The authors declare that the research was conducted in the absence of any commercial or financial relationships that could be construed as a potential conflict of interest.

## Publisher’s Note

All claims expressed in this article are solely those of the authors and do not necessarily represent those of their affiliated organizations, or those of the publisher, the editors and the reviewers. Any product that may be evaluated in this article, or claim that may be made by its manufacturer, is not guaranteed or endorsed by the publisher.
